# Heterogeneous Ribonucleoprotein A1 (hnRNPA1) Interacts with the Nucleoprotein of the Influenza a Virus and Impedes Virus Replication

**DOI:** 10.3390/v14020199

**Published:** 2022-01-20

**Authors:** Ramandeep Kaur, Jyoti Batra, Olga Stuchlik, Matthew S. Reed, Jan Pohl, Suryaprakash Sambhara, Sunil Kumar Lal

**Affiliations:** 1 School of Science, Monash University, Selangor 47500, Malaysia; ramanme24@gmail.com (R.K.); jyoti2111@gmail.com (J.B.); 2 Influenza Division, National Center for Immunization and Respiratory Diseases, Centers for Disease Control and Prevention, Atlanta, GA 30329, USA; hus0@cdc.gov (O.S.); iej4@cdc.gov (M.S.R.); hhe7@cdc.gov (J.P.); 3 Tropical Medicine & Biology Platform, Monash University, Selangor 47500, Malaysia

**Keywords:** protein-protein interactions, host-virus relationships, IAV replication, nucleoprotein, hnRNPA1

## Abstract

Influenza A virus (IAV), like other viruses, depends on the host cellular machinery for replication and production of progeny. The relationship between a virus and a host is complex, shaped by many spatial and temporal interactions between viral and host proteome, ultimately dictating disease outcome. Therefore, it is imperative to identify host-virus interactions as crucial determinants of disease pathogenies. Heterogeneous ribonucleoprotein A1 (hnRNPA1) is an RNA binding protein involved in the life cycle of many DNA and RNA viruses; however, its role in IAV remains undiscovered. Here we report that human hnRNPA1 physically interacts with the nucleoprotein (NP) of IAV in mammalian cells at different time points of the viral replication cycle. Temporal distribution studies identify hnRNPA1 and NP co-localize in the same cellular milieu in both nucleus and mitochondria in NP-transfected and IAV-infected mammalian cells. Interestingly, hnRNPA1 influenced NP gene expression and affected viral replication. Most importantly, hnRNPA1 knockdown caused a significant increase in NP expression and enhanced viral replication (93.82%) in IAV infected A549 cells. Conversely, hnRNPA1 overexpression reduced NP expression at the mRNA and protein levels and impeded virus replication by (60.70%), suggesting antagonistic function. Taken together, results from this study demonstrate that cellular hnRNPA1 plays a protective role in the host hitherto unknown and may hold potential as an antiviral target to develop host-based therapeutics against IAV.

## 1. Introduction

Influenza A is a zoonotic intracellular RNA virus that imposes a significant disease burden, with approximately 3–5 million infections annually worldwide. Currently, available intervention strategies are rendered ineffective due to the ever-evolving nature of the virus and the emergence of drug-resistant strains [1,2]. IAV, unlike other RNA viruses, replicates in the nucleus and consequentially gets access to a plethora of host factors facilitating its survival in the host [3,4]. Unraveling the physiological function of host proteins crucial for viral replication provides mechanistic insights into the intracellular viral lifestyle and disease pathogenesis that aids in developing novel antivirals [1,5]. 

NP forms the basic structural and functional unit of the viral ribonucleoprotein (vRNP) complex. Multiple studies have suggested that NP functions as an adapter protein at the center of host-virus interactions [6,7,8]. Further, NP manipulates various host signal transduction pathways by interacting with multiple host proteins like actinin-4, RNF 43, clusterin, AP 15, HSP 40, etc. [9,10,11,12,13]. Host RNA binding proteome (RBPome) is composed of evolutionarily conserved, abundantly expressed proteins that regulate various aspects of RNA metabolism and eventually affect gene expression [14,15,16,17].

Preliminary studies from our lab using high throughput proteomics approach; immunoprecipitation coupled with mass spectrometry (IP/LC-MS) to study the NP interactome in IAV infected A549 cells, identified hnRNPA1 as a plausible interacting partner of NP. hnRNPA1 is the most ubiquitously expressed member of the hnRNP A/B subfamily, known to regulate various aspects of RNA metabolism and gene expression [18,19]. Heterogeneous ribonucleoproteins (hnRNPs) are functionally the most well-characterized and evolutionarily conserved family of RNA binding proteins (RBPs), comprising of at least 40 different proteins from hnRNPA1-U [18,20]. Multiple hnRNP family members like hnRNP M, H1, A2B1, AB are reported to interact with IAV proteins and regulate viral replication [21,22,23,24]. Therefore, cellular RBPs are believed to be crucial factors shaping the host-virus dialogue [14]. Diverse binding affinities of hnRNPA1 to DNA, RNA, and protein shape this protein’s physiological role in the host, ranging from transcription, post-transcriptional modification, translation, binding affinity, and expression [19,25]. hnRNPA1 controls the expression of genes involved in crucial metabolic pathways and is implicated in a wide variety of cancers and neurodegenerative disorders [18,26,27,28]. Mutations of hnRNPA1 result in amyotrophic lateral sclerosis (ALS) and the syndrome multisystem proteopathy [29,30]. Unfortunately, what we know is the tip of the iceberg and needs subsequent investigation. 

There is mounting evidence that hnRNPA1, an RNA binding protein, interacts with many viral gene products and differentially regulates host-virus gene expression in viral infections [14,31]. It is shown that in Sindbis virus, EV-71, human rhinovirus (HRV), porcine epidemic diarrhea virus (PEDV) infections, hnRNPA1 promotes viral infection. In contrast, in others like human T-cell lymphotropic virus (HTLV-1) and hepatitis C virus (HCV), it abrogates viral infection, protecting the host [14,32,33,34,35,36]. However, there is a dearth of studies examining the role of hnRNPA1 in IAV infection. Here, endogenous hnRNPA1 was identified and validated as an interacting partner of NP in a mammalian cell system by co-immunoprecipitation. Temporal distribution studies identified that hnRNPA1-NP co-localize primarily in the nucleus and the mitochondria of the infected cell. We report that IAV infection enhanced hnRNPA1 protein expression in a dose-dependent manner. Most importantly, we have shown that hnRNPA1 overexpression attenuates NP gene expression at mRNA and protein level and abrogates viral replication. Concordantly, downregulation of hnRNPA1 enhanced NP gene expression and progeny virion production in IAV-infected cells. Our results demonstrate a novel role of human hnRNPA1 in the IAV replication. 

## 2. Materials and Methods

### 2.1. Cell Culture, Plasmids, and Antibodies

Human Embryonic Kidney (HEK293), Adenocarcinomic human alveolar basal epithelial cells (A549), Madin-Darby canine kidney (MDCK) were procured from Animal type tissue culture collection (ATCC, Manassas, VA, USA). Cells were maintained in Dulbecco’s minimal essential medium, high glucose (DMEM) (Gibco, Invitrogen, Waltham, MA, USA) supplemented with 10% fetal bovine serum (FBS), 1 mM Sodium hydrogen carbonate, 2 mM L-glutamine, and 1% penicillin-streptomycin (Gibco, Invitrogen, USA). Cells were incubated at 37 °C, 5% CO_2_ for 24–48 h. 

The NP gene of A/Puerto Rico/8/34 (PR8) H1N1 was cloned in pcDNA3.1 myc-His plasmid to be used as bait for immunoprecipitation studies (Invitrogen, USA). Human hnRNPA1 gene (NM_031157) cloned in pcDNA3.1+(C)-(K)-DYK vector was purchased from GenScript, USA. NP, hnRNPA1, β actin, and Vinculin were detected using specific antibodies. Anti-NP (ab66191) and anti-hnRNPA1 (ab4791) were purchased from Abcam (Abcam, Waltham, MA, USA). Anti-NP (9G8), anti-hnRNPA1 (4B10), anti-vinculin, anti-β actin antibodies were procured from Santa Cruz Biotechnology, USA (Santa Cruz, CA, USA). 

### 2.2. IAV Infection and Virus Infectivity Assay (Plaque Assay)

VR-95 (A/Puerto Rico/8/34 (H1N1) PR8) virus obtained from ATCC was used for infection, and virus titer was determined using MDCK cells, as described previously [13]. For infection studies, A549 cells were infected with *PR8* at a multiplicity of infection (MOI = 1) unless specified otherwise. After adsorption for 1 h, the unbound virus was removed by washing the cells with DMEM, and cells were incubated in DMEM media supplemented with 0.3% bovine serum albumin (BSA) and 0.1 μg/mL N-*p*-tosyl-1-phenylalanine chloromethyl ketone (TPCK) (Sigma Aldrich, St. Louis, MO, USA) to support infection. Cells were then incubated at 37 °C for 24 h in a 5% CO_2_ micro-environment.

MDCK cells cultured as monolayers (90–95% confluent) in DMEM complete media were incubated with 150–200 μL of virus inoculum at different serial dilutions in 0.3% BSA supplemented DMEM media, traversing from 10^0^–10^−4^ at 37 °C with 5% CO_2_, 1h. Briefly, MDCK cells were seeded in four 6 well plates at a seeding density of 0.325 × 10^6^ cells per mL and incubated at 37 °C for 22–24 h. Plates were rocked every 15 min to ensure uniform virus distribution for an hour. Mock infected cells were treated with minimal media supplemented with 0.3% BSA. Post-incubation, virus inoculum was removed and substituted with 0.6% low melting point agarose (LMP) (Life Technologies, Carlsbad, CA, USA) in L15 medium (2X L15, 1M HEPES, 50 μg/mL gentamycin, NaHCO_3_ & Pen Strep) (Hi-Media, Mumbai, India) supplemented with 2 μg/mL Trypsin-TPCK (Sigma-Aldrich, MO, USA) to support infection. Cells were incubated for 48 h at 37 °C. L15-agarose overlay was removed, and cells were stained with staining solution (1% crystal violet and methanol) for 30 min and washed with running water. After that, the staining solution was removed, and the number of plaques (plaque-forming unit (PFU)) was elucidated after drying, as described previously [13].

### 2.3. Plasmids and siRNA Transfection

Plasmid DNA transfections were performed using Lipofectamine 2000^TM^ (Invitrogen, Waltham, MA, USA) in HEK and or A549 cells maintained in serum and antibiotic-free basal DMEM media, following the manufacturer’s protocol. The culture was then replaced with DMEM media supplemented with 10% FBS and Penicillin streptomycin, 4–6 h post-transfection (Gibco, Invitrogen, USA) for indicated time points before processing. 

hnRNPA1 ON-TARGET plus SMARTpool siRNA was procured from Dharmacon, USA. Nontargeting control siRNA (NTC) (negative control) was procured from Santa Cruz, Biotechnology, USA. For siRNA-mediated transfection, A549 cells (80–90% confluency) were transfected with test or NTC control siRNA using RNAiMax^TM^ (Invitrogen, USA) in basal DMEM media for 6 h. Post-incubation, serum-free media was supplemented with complete media (10% FBS, 1% penicillin-streptomycin) and incubated for 24 h, 37 °C, 5% CO_2_. Cells were washed with 1X Phosphate buffer saline (PBS) followed by *PR8* infection at a multiplicity of infection (MOI = 1), unless specified otherwise, and incubated for 24 h, 37 °C, 5% CO_2_. Cell lysates were harvested at indicated time points and processed by immunoblotting. 

### 2.4. Protein Extraction, Western Blotting and SDS Polyacrylamide Gel Electrophoresis (PAGE)

Cell lysates were harvested and homogenized in 1X RIPA lysis Buffer (150 mM Sodium chloride, 50 mM Tris-Cl (pH-8.0), 1% nonyl phenoxypolyethoxylethanol (NP-40), 0.5% Sodium deoxycholate with 10 mM phenylmethylsulphonyl fluoride (PMSF), and protease inhibitor cocktail (PIC) (Roche, Basel, Switzerland) on ice. Total protein content in samples was deduced by standard Bradford assay (BioRad, Hercules, CA, USA) using BSA 2 mg/mL stock solution (Thermo Fischer Scientific, Waltham, MA, USA). Protein lysates (30–40 μg) were electrophoretically separated on an SDS PAGE (10–15%) and transferred using nitrocellulose membrane (Santacruz, Santa Cruz, CA, USA) via immunoblotting. Membranes were blocked using 2.5% *v*/*v* skim milk (Santacruz, USA) or 5% BSA prepared in Tris-buffered saline (TBS) (10 mM Tris HCl, pH 8, 150 mM NaCl) and 0.1% Tween-20 (Sigma Aldrich, USA), followed by primary and secondary horseradish peroxidase (HRP) conjugated IgG rabbit (Cell Signaling, Danvers, MA, USA) or IgG mouse antibody (Cell Signaling, USA). Chemiluminescent detection of proteins using Immobilon crescendo western blotting HRP substrate detection reagent (Merck Millipore, Burlington, MA, USA) or WESTAR Supernova (Cyanagen, Bologna BO, Italy), using the manufacturer’s protocol. Proteins bands were captured using the G: Box XX6 imaging system (Syngene, Cambridge, UK) and analyzed with Syngene gene tools software (Syngene, Cambridge, UK).

### 2.5. Co-Immunoprecipitation Assay

Cells were harvested in Lysis Buffer, washed (20 mM Tris-HCl pH 8.0, 10% glycerol, 137 mM NaCl, 0.5% NP-40, 2 mM EDTA, PIC (Roche, USA)) and subjected to co-immunoprecipitation. Cell extracts were mixed with appropriate antibodies and incubated at 4 °C, 10 rpm, and overnight. The following day, protein G Dynabeads^TM^ (Invitrogen, Life Technologies, USA) was mixed and rotated at 4 °C for 2–3 h. Post-incubation, beads were washed thrice with ice-cold 1X PBS, and protein-antibody complexes were eluted in 2X Laemmli Sample Buffer [13]. NP or hnRNPA1-immunoprecipitated proteins were detected by western blotting.

### 2.6. Immunofluorescence Imaging

A549 or HEK cells were seeded on coverslips in a 24-well culture plate. Cells were washed by 1X PBS and fixed by 4% paraformaldehyde (PFA) in PBS for 15 min and permeabilized using 1% Triton X-100 in PBS for 10 min (Sigma Aldrich, USA) post-infection or NP-transfection. The cells were then washed with 1X PBS and blocked with blocking buffer (2% BSA and 1X Phosphate buffer saline in 0.1% Tween 20 (PBST)) for 2 h, 40 rpm at room temperature (RT), followed by incubation with specific primary antibodies targeting either hnRNPA1 or NP in antibody dilution buffer (0.5% BSA, 0.3% Tween-20, and 1X PBS) for 2 h, 40 rpm at RT or 4 °C, overnight. After washing with 1X PBST cells thrice, 5 min each, cells were subsequently incubated with indicated Alexa Fluor (Abcam, USA) conjugated secondary antibodies for 2 h at RT. After washing with 1X PBST twice, the coverslips were mounted onto glass slides using the Prolong^TM^ Gold Antifade Mountant with DAPI (Thermo Fisher Scientific, USA) and incubated for 24 h at RT or 4 °C. Images were acquired by a fluorescence microscope (Nikon, Tokyo, Japan) or a Leica TCS SP5 II confocal imaging microscope (Leica, Wetzlar, Germany). The intensity-based quantification of confocal imaging data was done using the Leica application suite (LAS X 3.1.1) software. 

### 2.7. Subcellular Fractionation

For cellular fractionation assay, 1 × 10^6^ cells were seeded in a 100 mm cell culture dish and subjected to subcellular fractionation assay using Abcam cellular fractionation kit (ab17019, Abcam, USA) following the manufacturer’s protocol. Fractions hence obtained were subjected to immunoblotting and probed with anti-NP (Abcam, USA) and anti-hnRNPA1 (Santacruz, USA) antibodies. Vinculin (Santacruz, USA), Voltage-dependent anion-selective channel 1 (VDAC1) (Santacruz, USA), and LaminB1 (Santacruz, USA) were used as cytoplasmic, mitochondrial, and nuclear fraction controls to validate the purity of the fractions. 

### 2.8. RNA Extraction and Real-Time PCR (qRT-PCR) Analysis

Total RNA was extracted from cells after the specific treatment, at indicated time points, using RNeasy Mini Kit (Qiagen^TM^, Hilden, Germany) as per the manufacturer’s protocol (n = 9). Briefly, 1 μg of RNA was reverse transcribed using the ReverTraAce^®^ qPCR RT Master Mix with gDNA remover (Toyobo^TM^, Osaka, Japan) as per the manufacturer’s protocol. The quality of the cDNA synthesized was evaluated via Biodrop 260/280 nm (BioRad, Hercules, CA, USA). The resulting cDNA was diluted in a 1:10 ratio, and 150 ng of the cDNA was used as a template for SYBR green (Bioline, London, UK) based real-time PCR amplification reaction. The amplification was performed using the following conditions: one cycle of 95 °C for 3 min, 35–40 cycles of 95 °C for 30 s, and respective annealing for 45 s followed by melt curve analysis. Housekeeping genes like glyceraldehyde-3-phosphate dehydrogenase (GAPDH), was used as internal control, and the relative target gene fold change in NP and hnRNPA1 mRNAs was calculated using the delta-delta threshold cycle 2^−ΔΔCt^ method (in reference to the control) [37]. Statistical analysis was performed to compare the difference between two different treatment groups using the student’s *t*-test (two-tailed), respectively. 

The primers used for real-time based analysis are tabulated in Table 1.

### 2.9. Densitometry and Statistical Analysis

Western blots quantification (densitometry analysis) and qRT-PCR results are expressed as the mean ± S.D. from at least three independent experiments (n = 3), and statistical analysis was performed using two-tailed, student’s *t*-test by GraphPad Prism 9 (USA). 

## 3. Results

### 3.1. Human hnRNPA1 Interacts with IAV Nucleoprotein

**Figure 1:**
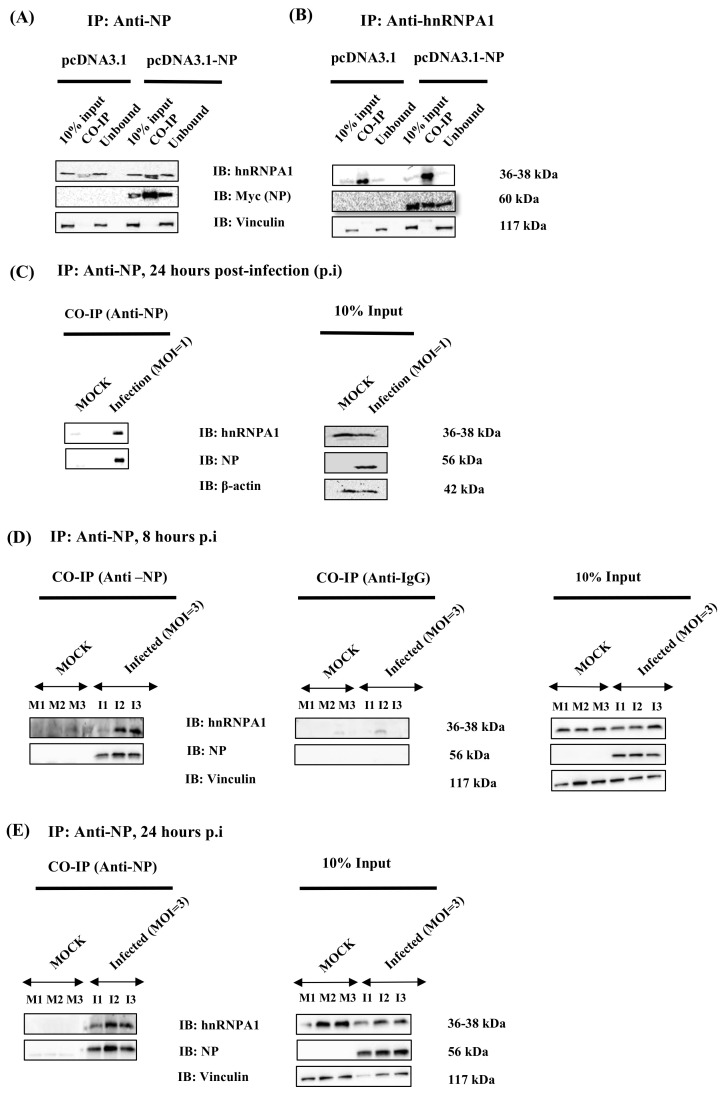
hnRNPA1 interacts with viral nucleoprotein (NP) in mammalian cells transfected with the NP expression plasmid and infected with A/Puerto Rico/8/34 virus H1N1 (*PR8*) by co-immunoprecipitation. HEK cells were transfected with either pcDNA3.1 (pcDNA3.1-myc/HisB) or pcDNA3.1-NP from *PR8* expression plasmids, 48 h post-transfection, cells were harvested, and co-immunoprecipitation (IP) was performed using (**A**) anti-NP (myc) antibody, (**B**) anti-hnRNPA1 antibody. Anti-NP (myc) antibody and anti-hnRNPA1 antibody were used to detect NP and hnRNPA1, respectively, by immunoblotting (IB). Vinculin was used as the loading control. (**C**), A549 cells were infected with *PR8* virus (MOI = 1) and harvested 24 h post-infection (p.i), followed by immunoprecipitation (IP) using anti-NP antibody. (**D**,**E**) A549 cells were infected with *PR8* virus at (MOI = 3) and harvested at designated time points, p.i, followed by immunoprecipitation (IP) using an anti-NP antibody (n = 3). Uninfected cells (Mock) were used as a control. IgG isotype control shows no nonspecific binding (**D**). anti-hnRNPA1, anti-NP, anti-Vinculin, and anti-β actin were used to detect the proteins by immunoblotting (IB). In the figure, Co-IP is the immunoprecipitated fraction, and unbound is the washed fraction. (**D**,**E**): M1, M2, M3 represent uninfected cells (biological triplicates, n = 3), and I1, I2, and I3 represent *PR8* infected cells (MOI 3) (n = 3).

Using high throughput proteomics techniques, immunoprecipitation coupled with liquid chromatography and mass spectrometry (IP-LC/MS), the interacting partners of Influenza A NP from *PR8* were screened in *PR8*-infected A549 cells. This preliminary screening identified hnRNPA1 as a putative interacting partner of NP, 8 h post-infection (p.i). (Data not shown). A co-immunoprecipitation assay was performed to confirm further and validate this interaction post-NP transfection and IAV infection (MOI 1, 3) in mammalian cells. To this end, HEK cells were transfected with pcDNA3.1-myc/HisB (vector control) or NP from H1N1 isolate cloned in pcDNA3.1-myc/HisB expression vector. The cell lysates were harvested 48 h post-transfection and subjected to co-immunoprecipitation assays and western blotting using anti-NP and/or anti-hnRNPA1 antibodies (Figure 1A–C). Myc-tagged expression of NP was confirmed using immunoblotting (Figure 1A,B). As shown in Figure 1A, immobilized anti-myc antibody (NP) precipitated a large amount of hnRNPA1 in pcDNA-3.1-myc/HisB-NP transfected cells than with the empty vector control (pcDNA3.1-myc/HisB). Similarly, a reciprocal IP, using anti-hnRNPA1 antibody followed by immunoblotting with anti-myc (NP) antibody, confirmed hnRNPA1-NP interaction (Figure 1B). These results demonstrate that IAV NP specifically associates with cellular hnRNPA1 in NP-transfected mammalian cells. 

After validating NP-hnRNPA1 in NP-transfected cells, we next sought to determine this interaction in IAV-infected cells. Briefly, A549 cells were infected with *PR8* at different multiplicities of infection (MOI), 1 and 3. Further, cells were harvested at indicated time points, p.i., and subjected to immunoprecipitation using anti-NP antibody. In line with our previous results, NP of *PR8* co-immunoprecipitated with human hnRNPA1 in A549 infected cells at 8 and 24 h p.i (n = 3) (Figure 1C–E), emphasizing its significance in the IAV life cycle. These results collectively confirm that human hnRNPA1 and NP are direct interacting partners.

### 3.2. Cellular hnRNPA1 Co-Localizes with Viral Nucleoprotein in NP-Transfected HEK Cells and IAV-Infected A549 Cells

**Figure 2:**
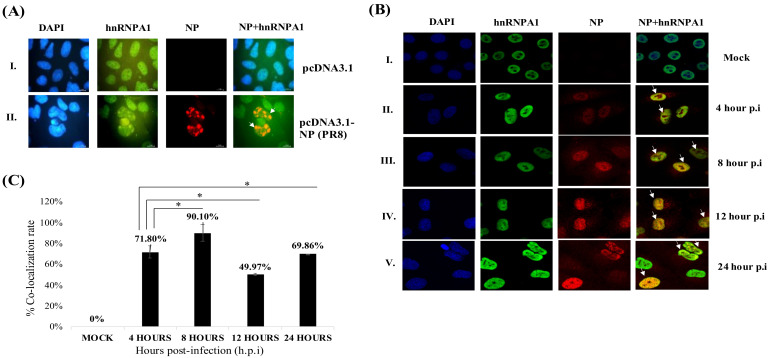
Co-localization of cellular hnRNPA1 and NP in the nucleus in NP transfected (**A**) and IAV infected (**B**) mammalian cells. (**A**) HEK cells were transfected with either empty vector, pcDNA3.1 (pcDNA3.1-myc/HisB), or pcDNA3.1-NP for 48 h and processed for immunofluorescence analysis. hnRNPA1 and NP were detected using anti-hnRNPA1 monoclonal antibody and Alexa flour 488 conjugated secondary antibody (green); NP polyclonal antibody and Alexa flour 594 conjugated secondary antibody (red), respectively. Arrows indicate co-localization of NP and hnRNPA1 in NP transfected cells. Panel I shows pcDNA3.1 transfected (vector control), and Panel II depicts pcDNA3.1-NP transfected cells. Images were acquired using an immunofluorescence microscope (Nikon, Japan). (**B**) A549 cells were either uninfected (Mock) (Panel I) or *PR8* infected (MOI = 1) (Panel II, III, IV, V) and fixed with 4% paraformaldehyde at indicated time points, p.i. hnRNPA1 and NP were detected using anti-hnRNPA1 antibody and Alexa fluor 488 conjugated secondary antibody (green); anti-NP polyclonal antibody, followed by Alexa fluor 594 conjugated secondary antibody (red), respectively. Cells were mounted using ProLong^TM^ Gold Antifade Mountant with DAPI (blue) and processed for confocal microscopy (63X). Arrows indicate co-localization of NP and hnRNPA1 in IAV infected cells. (**C**) Intensity-based quantification of confocal data was performed using LASX 3.1 software (Leica Microsystems, Wetzlar, Germany). Co-localization rate (%) (y-axis) of NP and hnRNPA1 at different times post-infection (p.i) (x-axis) was extrapolated. Co-localization % was computed using Pearson’s correlation (+1–(−1)) (+1: perfect co-localization and −1: perfect exclusion) and Overlap Coefficient (0–1) (1: perfect co-localization and 0: No co-localization) by LASX 3.1 using the intensity-based quantification feature. Briefly, 10–13 z-stack slices per image per time point were selected, and a co-localization rate was generated using Pearson’s correlation coefficient and Overlap Coefficient for further processing (n = 3). Statistical analysis was performed using one-way ANOVA and post-hoc Tukey test, * indicates that each time point data is significant with respect to each other.

Cellular translocation of hnRNPA1 in response to external stimuli like stress, osmotic shock, or viral infections is well known; however, its expression and temporal distribution in IAV infection are rather elusive. After validating hnRNPA1 and NP as direct interacting partners, we next performed kinetics studies to determine the primary site of hnRNPA1 and NP interaction in mammalian cells. This was investigated by adopting two approaches: immunostaining by confocal microscopy and subcellular fractionation. In the first approach, HEK cells were transfected either with pcDNA3.1-myc/HisB (vector control) or pcDNA3.1-myc/HisB-NP plasmid to induce the expression of viral NP in mammalian cells transiently. Cells were fixed with 4% PFA, 48 h post-transfection, and processed for immunostaining by incubating with NP and hnRNPA1 primary antibodies and Alexa fluor 594 (NP) and Alexa fluor 488 (hnRNPA1) tagged secondary antibodies. To stain the nucleus, 4′,6-diamidino-2-phenylindole (DAPI) was used (Figure 2A). As shown in Figure 2A, Panel II, NP, and hnRNPA1 co-localized predominantly in the nucleus in NP transfected cells (48 h). pcDNA3.1-myc/HisB transfected cells (control) showed no co-localization (Figure 2A, Panel I).

Similar results were seen in *PR8* infected A549 cells by confocal microscopy. As shown in Figure 2B, NP and hnRNPA1 co-localized predominantly in the nuclear sub-compartment at different time points, p.i (Figure 2B, Panel II, III, IV, and V). The data collected from three independent regions of interest are represented graphically, wherein Pearson’s correlation coefficient and Overlap coefficient was used to measure the degree of co-localization hnRNPA1 and NP (Figure 2C). Quantification of confocal data in IAV-infected A549 cells suggested maximal co-localization (Pearson’s correlation coefficient, 0.701; Overlap coefficient, 0.729) in the nucleus in the following pattern: 8 h > 4 h > 24 h > 12 h in IAV-infected A549 cells (Figure 2C). As IAV replicates in the nucleus, NP and hnRNPA1 co-localization hints that human hnRNPA1 may influence IAV replication.

**Figure 3:**
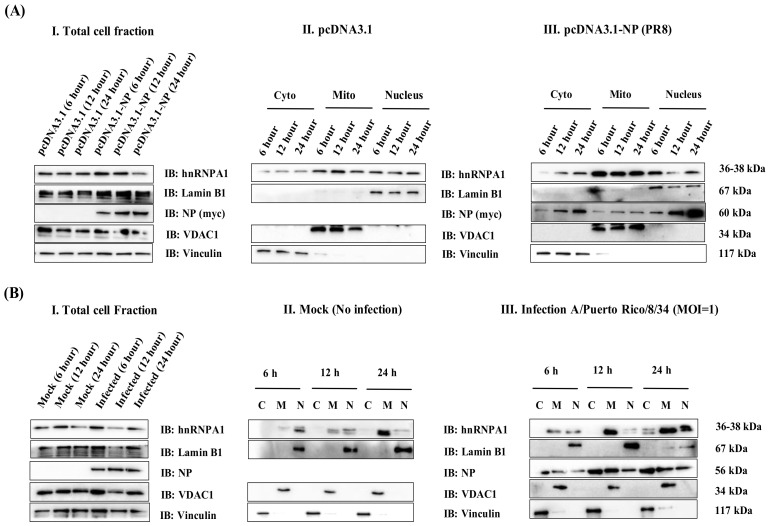
NP of IAV co-localizes with cellular hnRNPA1, primarily in the nucleus and mitochondrial compartment in pcDNA3.1-NP-transfected (**A**) and IAV-infected cells (**B**). (**A**) HEK cells were transfected with either vector control, pcDNA3.1 (pcDNA3.1-myc/HisB) (Panel II) or pcDNA3.1-NP (pcDNA3.1-myc/HisB-NP) plasmids (Panel III). Cell lysates were harvested at the designated time points (6, 12, 24 h), post-transfection, and subjected to subcellular fractionation, followed by immunoblotting. (**B**) A549 cells were either uninfected (Mock) (Panel II) or infected with *PR8* (Panel III) at an MOI of 1 and subjected to subcellular fractionation at designated time points p.i (6, 12, 24 h) and processed for immunoblotting. Anti-vinculin, anti-VDAC1, and anti-laminB1 were used as controls for cytoplasmic, mitochondrial, and nuclear fractions. hnRNPA1 and NP were detected using anti-hnRNPA1 and anti-NP primary antibodies. Cyto; C: cytoplasmic fraction, Mito; M: mitochondrial fraction, and N: nucleus.

Viral infections are known to regulate the cellular localization of hnRNPA1; therefore, to ascertain and confirm the enrichment of hnRNPA1 and IAV NP in various subcellular compartments, cellular fractionation assay was performed using Abcam fractionation kit (ab 109719) following the manufacturer’s protocol in pcDNA3.1-myc/HisB-NP transfected HEK cells and *PR8*-infected cells (MOI = 1). hnRNPA1 expression was concentrated in the mitochondrial and nuclear sub-compartments at all the time points investigated in both pcDNA3.1-myc/HisB-NP-transfected cells (Figure 3A, Panel II and III) and *PR8*-infected cells (Figure 3B, Panel II and III). In uninfected cells, hnRNPA1 was diffusely distributed across all fractions while IAV infection upregulated endogenous hnRNPA1 expression compared to its uninfected control (Mock) (Figure 3B, Panel III). On the other hand, NP was diffusely distributed across all the compartments with an overall increase in expression at later time points (12 and 24 h) of infection (Figure 3B, Panel III). As seen in this study, enhanced NP expression and cytoplasmic translocation may be attributed to the synthesis of viral proteins, as previously reported [11,39]. In addition, at later stages, hnRNPA1 and NP co-localized in all the fractions, with maximal co-localization in the nucleus and mitochondria. Uninfected cells (Mock) showed no co-localization (Figure 3B, Panel II). hnRNPA1 is a nuclear protein known to alter its subcellular location in response to viral infections, mitochondrial localization of this RBP in IAV infection is very interesting. Based on the preceding findings, we may deduce that hnRNPA1-NP co-localizes in NP transfected and IAV infected cells at early and late stages of infection, further supporting our previous observation. Thereby implying that the site of hnRNPA1-NP interaction may be in the nucleus and mitochondria of the cell.

### 3.3. Influenza Infection Enhances hnRNPA1 Protein Expression in IAV-Infected A549 Cells

**Figure 4:**
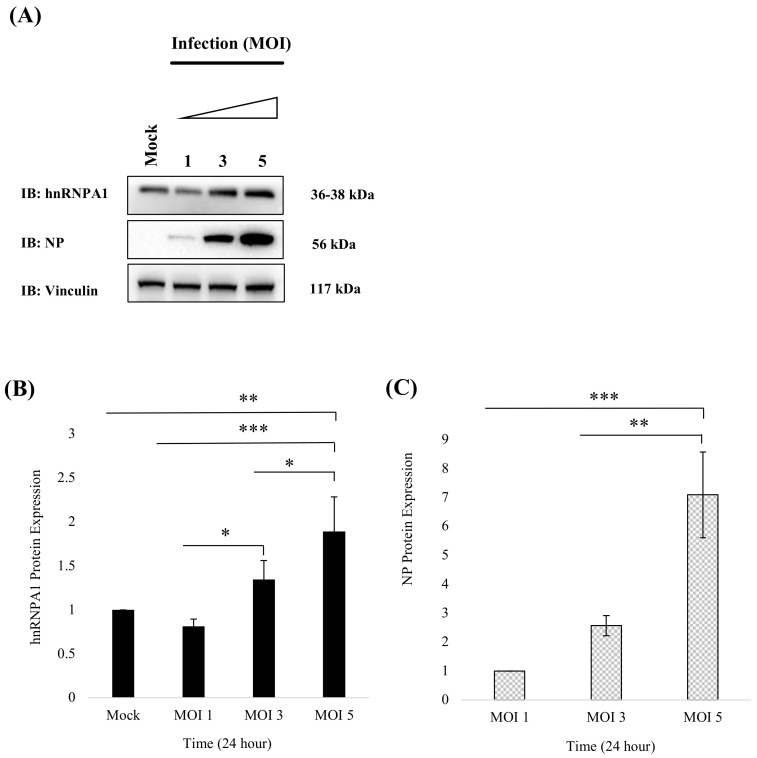
Increasing viral load enhances cellular hnRNPA1 protein expression. (**A**) A549 cells were either uninfected (Mock) or infected with (*PR8*) virus at varying MOI (1, 3, 5). Cell lysates were harvested 24 h p.i in RIPA Buffer, and 30 μg of the sample were subjected to immunoblotting to detect hnRNPA1, NP, and vinculin using specific antibodies. (**B**) Relative fold change in the expression of hnRNPA1 protein was deduced by densitometry analysis using Syngene gene tools analysis software (Syngene, Cambridge, UK) and relative hnRNPA1 protein levels normalized against vinculin and, uninfected sample group (Mock) were plotted (n = 3). (**C**) Relative fold change in the expression of NP protein was deduced by densitometry analysis using Syngene gene tools analysis software (Syngene, Cambridge, UK), and relative NP protein levels normalized against vinculin MOI 1 sample group were extrapolated. Results shown in (**B**,**C**) represent mean ± S.D. from three independent experiments (n = 3). Statistical significance was determined using one-way ANOVA with post-hoc Tukey test *, *p* < 0.05, **, *p* < 0.01, ***, *p* < 0.001.

Based on our findings that NP and hnRNPA1 interact and co-localize in the same cellular compartments, we investigated if Influenza infection influenced hnRNPA1 protein expression in a dose-dependent manner. To achieve this, A549 cells were infected with *PR8* at MOI 1, 3, 5, and relative hnRNPA1 protein expression was studied, 24 h p.i in A549 cells (Figure 4A,B). As shown in Figure 4A,C, increasing viral load (MOI 1, 3, 5) positively regulated NP expression (n = 3), *p* < 0.05. Likewise, hnRNPA1 expression exhibited a significant increase with increasing MOI with up to 34.4%, 89% increment at MOI 3 and 5, respectively (n = 3), *p* < 0.05 (Figure 4A,B). Thereby hinting that increasing viral load concurrently upregulates hnRNPA1 expression in IAV-infected cells. This is in line with previous reports wherein viruses adopt diverse strategies to regulate the expression of crucial host proteins for their benefit [10,13,39].

### 3.4. RNAi-Mediated Knockdown of hnRNPA1 Enhances Nucleoprotein Gene Expression

**Figure 5:**
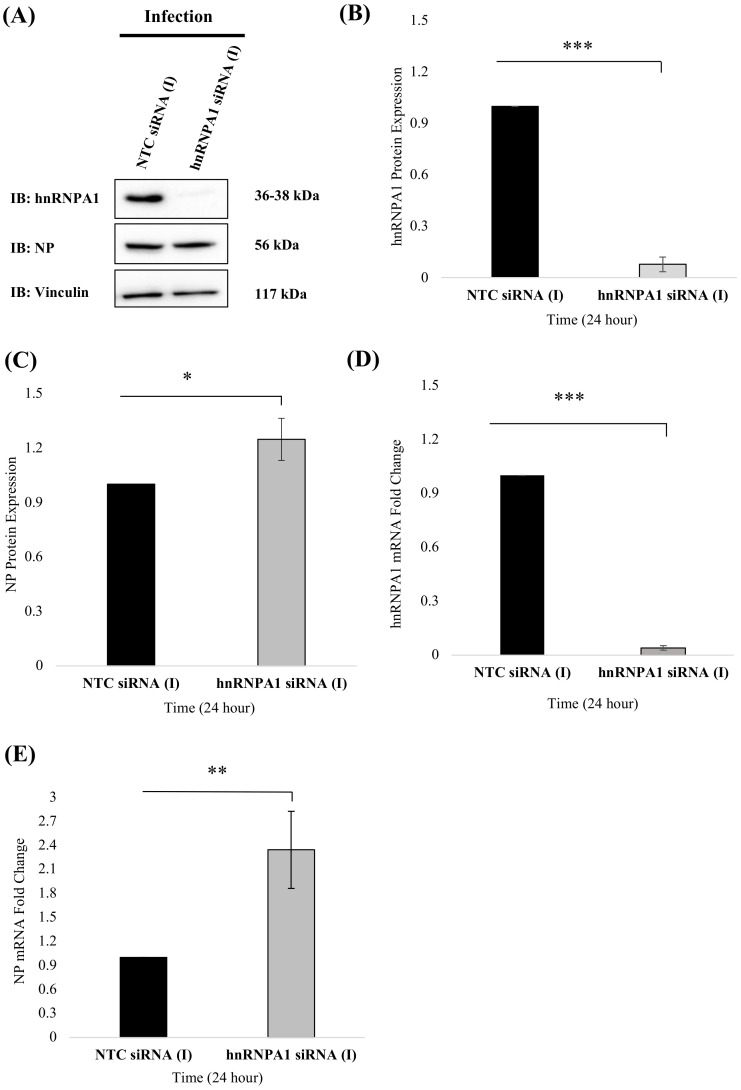
siRNA-mediated knockdown of hnRNPA1 enhances NP gene expression and viral replication in A549 cells. (**A**) A549 cells were transfected with non-targeting control (NTC) siRNA pool or hnRNPA1 specific siRNA pool for 24 h. At 24 h post-transfection, cells were either uninfected (Mock) or infected with *PR8* at an MOI of 1. Cell lysates were harvested in RIPA buffer, and 30 μg of the sample was analyzed by SDS PAGE and western blotting using anti-NP and anti-hnRNPA1 antibodies (n = 3). Vinculin was used as a loading control. (**B**,**C**) The fold change in expression levels of hnRNPA1 (**B**) and NP (**C**) protein was deduced by densitometry analysis using Syngene gene tools analysis software (Syngene, Cambridge, UK) and plotted. Results shown in (**B**,**C**) represent mean ± S.D. from three independent experiments (n = 3). Statistical significance was determined using a nonparametric, two-tailed student’s *t*-test. *, *p* < 0.05, **, *p* < 0.01, ***, *p* < 0.001. (**D**,**E**) Total cellular RNA was isolated, and relative mRNA expression of (**D**) hnRNPA1 and (**E**) NP was deduced using specific primers by qRT-PCR (n = 9). Briefly, A549 cells were transfected with non-targeting control siRNA (NTC) (NTC siRNA (I)) or hnRNPA1 siRNA pool (hnRNPA1 siRNA (I)) for 24 h, followed by infection with *PR8* at an MOI of 1. Data shown in (**D**,**E**) represent mean ± S.D. from three independent experiments (n = 9). Statistical significance between control and treated sample groups was determined using nonparametric, two-tailed student’s *t*-test *, *p* < 0.05, **, *p* < 0.01, ***, *p* < 0.001. NTC siRNA (I): NTC control siRNA treated *PR8* infected cells and hnRNPA1 siRNA (I): hnRNPA1 siRNA treated *PR8* infected cells.

Next, we examined the consequences of hnRNPA1 downregulation on NP gene expression in infected cells. hnRNPA1-specific small interfering RNAs (siRNAs) were used to knockdown constitutively expressed hnRNPA1 in mammalian cells. A total 30 nM concentration of hnRNPA1 siRNA pool was used to achieve optimal silencing in A549 cells (Figure 5). hnRNPA1 expression was modulated via small interfering RNA (siRNA) against hnRNPA1 in *PR8*-infected A549 cells. hnRNPA1 protein levels were drastically reduced in cells transfected with hnRNPA1 SMART pool siRNA compared to the NTC siRNA control (Figure 5A). The efficiency of hnRNPA1 knockdown was validated by western blotting as significant hnRNPA1 depletion (~90%) was observed in hnRNPA1 siRNA treated cells in comparison to NTC siRNA treated control (n = 3), *p* < 0.05 (Figure 5A,B). Further, hnRNPA1 siRNA treated cells exhibited enhanced NP protein expression (n = 3), *p* < 0.05 (Figure 5A,C).

As hnRNPA1 depletion enhanced NP protein expression, we anticipated that NP mRNA expression would also be impacted. To test this hypothesis, NP mRNA levels were monitored in hnRNPA1 depleted, IAV infected cells via quantitative real-Time PCR (qRT-PCR). Briefly, total RNA isolated from A549 cells treated with either hnRNPA1 specific siRNA or NTC siRNA, followed by infection with *PR8* virus (MOI = 1) and subjected to qRT-PCR analysis (Figure 5D,E). hnRNPA1 mRNA levels were significantly downregulated (~97.12%) in hnRNPA1 siRNA treated cells (n = 9), *p* < 0.05 (Figure 5D). On the contrary, NP mRNA levels showed significant upregulation (~2.3 folds) in hnRNPA1 silenced, IAV-infected cells, 24 h p.i (n = 9), *p* < 0.05 (Figure 5E). NTC siRNA treated, IAV infected cells were used as a negative control. This alteration in NP gene expression in hnRNPA1 depleted, IAV infected cells implies an antagonistic relationship between hnRNPA1 and NP.

### 3.5. hnRNPA1 Overexpression Reduces NP Protein and mRNA Expression In Vitro

**Figure 6:**
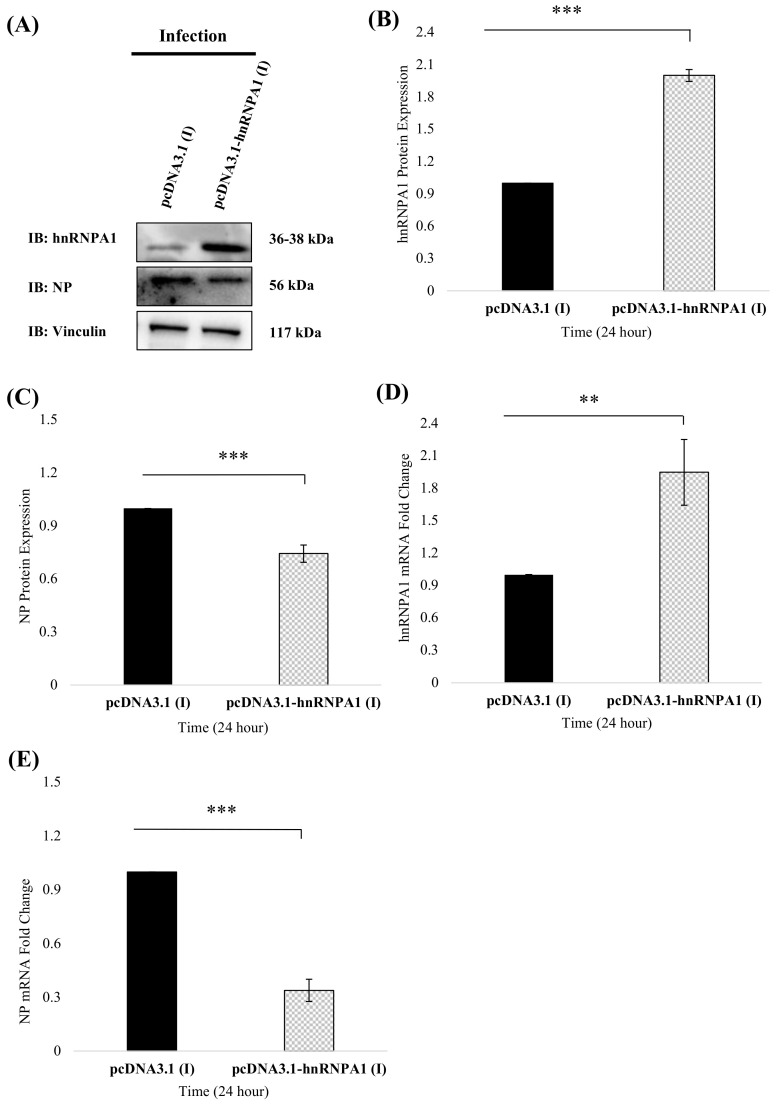
hnRNPA1 overexpression suppresses NP protein and mRNA expression and impedes viral replication. A549 cells were transfected with either pcDNA3.1 (vector control) or pcDNA3.1-hnRNPA1 for 24 h and infected with *PR8* at an MOI of 1. 24 h later, cells were harvested in RIPA buffer and subjected to immunoblotting. (**A**) Relative expression of NP and hnRNPA1, post-hnRNPA1 overexpression in *PR8* at an MOI of 1 infected A549 cells was deduced via immunoblotting using anti-NP and anti-hnRNPA1 antibodies (n = 3). Vinculin was used as a loading control. The fold change in expression levels of hnRNPA1 (**B**) and NP (**C**) protein was deduced by densitometry analysis using Syngene gene tools analysis software (Syngene, Cambridge, UK) and plotted. Results shown in (**B**,**C**) represent mean ± S.D. from three independent experiments (n = 3). Statistical significance was determined using a nonparametric, two-tailed student’s *t*-test. **, *p* < 0.01, ***, *p* < 0.001. (**D**,**E**) Total cellular RNA was isolated, and relative mRNA expression of (**D**) hnRNPA1 and (**E**) NP was deduced using specific primers by qRT-PCR (n = 9). A549 cells were transfected with either pcDNA3.1-hnRNPA1 or pcDNA3.1 expression (vector control) constructs for 24 h, followed by *PR8* infection at an MOI of 1. At 24 h, p.i, the total cellular RNA was isolated, and relative mRNA expression of NP and hnRNPA1 was deduced using specific primers by qRT-PCR. The data in (**D**,**E**) show mean ± S.D. from at least three independent experiments (n = 9). Statistical significance was determined using nonparametric, two-tailed student’s *t*-test. **, *p* < 0.01, ***, *p* < 0.001. pcDNA3.1 (I): pcDNA3.1 transfected, *PR8* infected A549 cells and pcDNA3.1-hnRNPA1 (I): pcDNA3.1-hnRNPA1 transfected and *PR8* infected A549 cells.

Because hnRNPA1 silencing enhanced NP gene expression, we next sought to determine if overexpressing hnRNPA1 in IAV infection exhibited an opposite effect. For this, hnRNPA1 expression was transiently upregulated by transfection with human hnRNPA1 cloned in a mammalian expression vector; pcDNA3.1-hnRNPA1, followed by infection with *PR8* virus (MOI = 1). Briefly, A549 cells were transfected with either pcDNA3.1 control plasmid or pcDNA3.1-hnRNPA1 plasmid, followed by IAV infection (Figure 6). Cell lysates harvested 24 h; p.i were subjected to western blotting. hnRNPA1 expression was significantly upregulated in IAV-infected cells (n = 3), *p* < 0.005 (Figure 6A). Concordantly, cells with enhanced hnRNPA1 expression showed a significant decline in NP protein levels (n = 3), *p* < 0.005 in comparison to pcDNA3.1 transfected cells (negative control), suggesting that enhancing hnRNPA1 cellular levels suppresses NP protein expression in IAV infection (Figure 6A,B). 

Similarly, NP mRNA expression was studied under the influence of hnRNPA1 upregulation in IAV infected cells via qRT-PCR. hnRNPA1 mRNA levels were significantly enhanced (~2 folds) in pcDNA3.1-hnRNPA1 transfected cells (n = 9), *p* < 0.005 (Figure 6D). As expected, NP mRNA expression was attenuated by ~3 folds in hnRNPA1 upregulated, IAV-infected cells with respect to control (n = 9), *p* < 0.001 (Figure 6E). These results corroborate our previous findings and emphasize the role of human hnRNPA1 in influencing viral NP gene expression.

### 3.6. Human Heterogeneous Ribonucleoprotein A1 Expression (HNRNPA1) Impacts IAV Replication In Vitro

**Figure 7:**
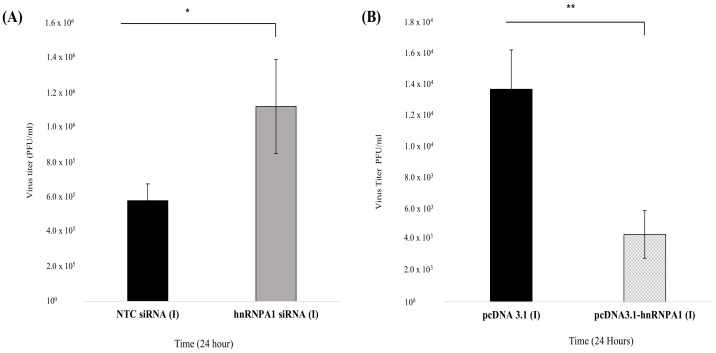
hnRNPA1 regulates viral replication post-hnRNPA1 knockdown and overexpression in IAV infected A549 cells. (**A**) Effect of hnRNPA1 silencing on progeny virion production in IAV-infected A549 cells. A549 cells were treated with NTC siRNA or hnRNPA1 siRNA pool, followed by infection with *PR8* virus at an MOI of 1. Aliquots of supernatants collected after 24 h were used to determine viral titers (PFU/mL) by plaque assay. Virus titers (PFU/mL) in NTC siRNA- or hnRNPA1 siRNA-transfected cells were calculated and plotted (10^−3^ dilution was used to enumerate plaques). (**B**) Effect of hnRNPA1 over-expression on viral replication. Briefly, A549 cells were transiently transfected with either vector control, pcDNA3.1, or pcDNA3.1-hnRNPA1 expression construct for 24 h, followed by infection with *PR8* at an MOI of 1, 24 h later. Virus titer (PFU/mL) in supernatants of pcDNA3.1- and pcDNA3.1-hnRNPA1-transfected cells collected 24 h p.i. were calculated and plotted (10^−1^ dilution was used for enumeration of plaques. Data shown in (**A**,**B**) represent mean ± S.D. from at least three independent experiments (n = 3). Statistical significance was determined using a nonparametric, two-tailed student’s *t*-test. *, *p* < 0.05, **, *p* < 0.01, respectively.

Viral infections are known to regulate the expression of key host proteins to benefit their replication. To investigate this aspect, we examined viral replication after transient hnRNPA1 overexpression (plasmid transfection) or knockdown (specific siRNA mediated) in A549 cells (Figure 7A,B). To study the consequence of hnRNPA1 depletion on viral replication, A549 cells were transfected with NTC siRNA or hnRNPA1 siRNA pool and infected with; *PR8* at a MOI = 1. Cell supernatants collected 24 h p.i were used to measure virus titers in MDCK cells via plaque assay (Figure 7A). In line with our previous observations, there was a steep increase of ~93.82% in viral replication as reflected by increased virus titer (PFU/mL), post-hnRNPA1 siRNA treatment in comparison to NTC siRNA treated control population (n = 3), *p* = 0.01 (Figure 7A). Similarly, the effect of transient hnRNPA1 overexpression on progeny virion production was also deduced. Briefly, A549 cells were transfected with either control plasmid (pcDNA3.1) or pcDNA3.1-hnRNPA1 expression plasmid for 24 h, followed by infection with *PR8* at a MOI = 1 (Figure 7B). Supernatant collected 24 h p.i was analyzed for viral growth by plaque assay in MDCK cells, as described previously [13] (Figure 7B). As shown in Figure 7B, enforced hnRNPA1 expression in IAV-infected cells reduced virus titer by ~60.70%, impeding progeny virion production, suggesting antiviral function (n = 3), *p* = 0.006 (Figure 7B).

Collectively, these results advocate that human hnRNPA1 is a negative regulator of virus replication and has a protective role in IAV infection.

## 4. Discussion and Conclusions

The limited coding capacity of viruses, including IAV, depends on the host cellular systems for propagation and survival, paving the way for a plethora of interactions between viral and host cellular compartments in infected cells [1]. Virus-host interactions are the foundation of communication between the host and pathogen, which dictate the initiation and outcome of infection. Therefore, a comprehensive understanding of the host factors interacting with the viral machinery is crucial to understanding viral pathogenesis. Owing to the limited efficacy of currently available virus-derived treatments targeting host proteins to thwart viral infection is a promising approach. Transcriptional profiling of virus-infected cells and genome-wide screens highlighted the plausible involvement of hnRNPA1 in viral infections [14,19,30,40,41,42]. However, its role in Influenza infection is poorly defined. In this study, utilizing co-immunoprecipitation, we confirmed and validated that NP of IAV interacts with human hnRNPA1 in NP-transfected and IAV-infected mammalian cells.

Viral infections impact the host microenvironment by modulating the expression and localization of essential cellular proteins, including hnRNPA1, consequently affecting disease pathogenesis [5,21,40,42,43,44,45]. Temporal distribution studies identified increased hnRNPA1-NP co-localization in the nuclear and mitochondrial compartments in NP-transfected and IAV-infected cells, thereby enhancing the possibility of interaction between the two. Mitochondrial accumulation of Influenza A vRNP is well documented as it is associated with the induction of interferon (IFN) and antiviral immune signaling [46,47,48,49,50]. On the other hand, hnRNPA1 is enriched in the nuclear and cytosolic fraction in response to various internal and external stimuli [51]. The present study provides evidence about the mitochondrial localization of hnRNPA1. Considering the recent studies highlighting the role of mitochondria in regulating innate immune signaling in response to viral infections and the negative effect of hnRNPA1 on IAV replication characterized in this study [48,52], it can be speculated that hnRNPA1-NP co-localization in the mitochondria may be a strategy adopted by IAV to regulate mitochondrial antiviral innate immune responses to facilitate infection. However, these possibilities need to be examined in detail. 

**Figure 8:**
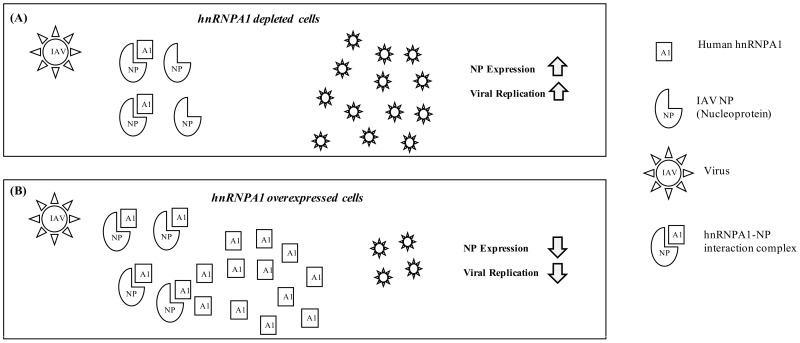
The proposed model depicts the role of hnRNPA1 in IAV infection. hnRNPA1 modulation is proposed to affect NP gene expression and viral replication. hnRNPA1 interacts with IAV NP in mammalian cells. IAV infected cells with reduced hnRNPA1 expression exhibit enhanced NP expression and viral replication (**A**). Contrastingly, IAV infected cells with enhanced hnRNPA1 levels show reduced NP expression and abrogated viral replication (**B**).

hnRNPA1 interacts with multiple viral gene products and influences viral replication in other viruses like HIV, HTLV-I, and PEDV [14]. We have also shown that in an IAV micro-environment, hnRNPA1 knockdown significantly enhanced NP gene expression and promoted viral replication in A549 cells, suggesting a possible role of hnRNPA1 in the IAV life cycle. Contrastingly, hnRNPA1 overexpression significantly reduced NP gene expression. The decline in NP protein and mRNA levels correlated with a reduced number of plaques observed in hnRNPA1 overexpressed, IAV-infected cells, highlighting its antiviral potential. IAV is known to induce and promote G0/G1 cell cycle arrest to facilitate transcription and translation of viral gene products [53,54]. hnRNPA1 depletion is also reported to enhance cell survival and promote G0/G1 cell cycle arrest in lung cancer [55], thereby explaining increment in the enumerated plaques in hnRNPA1 depleted, IAV-infected cells. The increment in virus titer, post-hnRNPA1 silencing further strengthens our hypothesis that hnRNPA1 depletion may alter the host cellular landscape, making it conducive for viral replication, evident by the increased number of plaques, whereas hnRNPA1 overexpression exerted an equally opposite effect, hence the consequent decline in virus titer depicted in Figure 8. In addition to Influenza A, hnRNPA1 has also been reported to have an antiviral role in HTLV-I and HCV infections [14].

The findings from our study unravel a novel role of the host RNA binding protein, hnRNPA1, in regulating the expression of viral nucleoprotein gene products (the primary factor that dictates viral replication and disease pathogenesis) and IAV replication. The protective role of hnRNPA1 in IAV infection sheds light on the unforeseen aspects of hnRNPA1 functionality and substantiates the role of NP as an adapter protein for host-virus cross-talk. Future studies are needed to address the molecular mechanisms by which hnRNPA1-NP interaction regulates viral gene expression, antiviral responses, and IAV replication which may aid in the development of next-generation therapeutics.

## Figures and Tables

**Table 1:** qRT-PCR primers and sequence details used in this study.

Target Gene	Primer Name	Sequence (5′–3′)	References
Human hnRNPA1	Forward	TGGACCCATGAAGGGAGGAA	
	Reverse	GCAAAGTATTGGCCTCCACC	
Human GAPDH	Forward	TCACTGCCACCCAGAAGACTG	
	Reverse	GGATGACCTTGCCCACAGC	
NP mRNA	Forward	TGTGTATGGACCTGCCGTAGC	[38]
	Reverse	CAATCCACACCAGTTGACTCTTG	[38]

## Data Availability

Data is presented within the article.
